# Odonto-Periosteal Tumor Simulating Pulp—Fungoid

**Published:** 1884-03

**Authors:** John S. Smith

**Affiliations:** Lancaster, PA.


### Odonto-Periosteal Tumor Simulating Pulp—Fungoid.

BY JOHN S. SMITH, D.D.S., OF LANCASTER, PA.

Miss K., a lady aged 21 years, presented herself for treatment October 12, 1883.

She had long suffered from neuralgia, confined principally to the right side of the face, and particularly along the course of the inferior maxillary nerve.

Examination revealed caries of the teeth. Several had died, and there were suppurating pulps in others which were exposed and congested.

When making the examination, my attention was arrested by seeing a tumor the size of a large bean, which completely filled a carious cavity of the first permanent molar tooth on the lower jaw. The crown of the tooth was broken in places nearly to the line of the gum, and the tumor overlapped the rough edges. This caused the growth to present an angry and inflamed appearance. There was considerable pain when pressure was made to the alveolar process corresponding to the tooth.

The growth was over three years old. It had not been troublesome until within the past year, during which time it had steadily grown worse, and this, and the painful condition of her other teeth already mentioned, drove the lady to seek surgical aid.

*Diagnosis.*—What is this growth? To all appearance it looks not unlike the pulp-fungus which has its origin in the bulbous portion of the tooth-pulp, and could be readily mistaken for a fungus of that expression. In the latter condition the removal of the tooth would be alone sufficient for a cure. The removal of the tooth would also remove the tumor.

We find by a careful examination with a probe that the fangs of the molar have been suppurated, and that the probe passes through into the alveoli; in fact, the fangs seem to be imbedded in the stroma. The fangs are loose, and portions of the alveolar process have been absorbed, so that the probe can readily be passed through it.

That the tumor is an outgrowth of that aspect of the odonto-alveolar membrane which adjoins the bone, is beyond doubt. The growth has passed through an opening between the roots of the tooth, making its appearance as above described.

We will, therefore, exclude pulp-fungus, which has its origin within the stroma of the dental pulp, which condition is perfectly innocent and benign in its nature.

The tumor was a form of epulide simulating, as location is concerned, the pulp-fungus, but a portion of the jaw was found implicated. It had involved it. In this case the simple removal of the tooth would not effect a cure.

*Treatment.*—The patient was placed in a Morrison dental chair, in a recumbent position, and etherized, Dr. D. R. Summy and Martin H Musser assisting. The fangs were quickly removed. The engine burs were then made to complete the operation by cutting down that portion of the alveolus and bone involved; the gums were not cut away by the operation, but left intact on the outer and inner borders, this being preferable to removal, as it is a means of retaining the after-dressing when found necessary. The hemorrhage was easily controlled with phenol sodique and tannic acid on cotton packed into the cavity.

In two weeks the wound was nicely granulated, and there has been no return of the tumor.

The other diseased teeth were subsequently treated and properly filled, and the neuralgic pains have entirely disappeared.

---

Dr. Philip Leidy recommends oil of citronella (two drops to the drachm) to disguise the odor of iodoform.

---

How to take Tincture of Iron.—To disguise the taste of tincture of iron, Dr. Haner recommends that tincture of the sesquichloride of iron be mixed with simple syrup, and then with milk. This mixture will not affect the teeth, nor will the styptic taste be apparent.

